# Poly(ADP-Ribose) Glycohydrolase (PARG) vs. Poly(ADP-Ribose) Polymerase (PARP) – Function in Genome Maintenance and Relevance of Inhibitors for Anti-cancer Therapy

**DOI:** 10.3389/fmolb.2020.00191

**Published:** 2020-08-28

**Authors:** Daniel Harrision, Polly Gravells, Ruth Thompson, Helen E. Bryant

**Affiliations:** Academic Unit of Molecular Oncology, Sheffield Institute for Nucleic Acids (SInFoNiA), Department of Oncology and Metabolism, University of Sheffield, Sheffield, United Kingdom

**Keywords:** poly(ADP-ribose)polymerase (PARP), poly(ADP-ribose)glycohydrolase (PARG), synthetic lethality, chemosensitization, radiosensitization, cancer

## Abstract

Poly(ADP-ribose) polymerases (PARPs) are a family of enzymes that catalyze the addition of poly(ADP-ribose) (PAR) subunits onto themselves and other acceptor proteins. PARPs are known to function in a large range of cellular processes including DNA repair, DNA replication, transcription and modulation of chromatin structure. Inhibition of PARP holds great potential for therapy, especially in cancer. Several PARP1/2/3 inhibitors (PARPi) have had success in treating ovarian, breast and prostate tumors harboring defects in the homologous recombination (HR) DNA repair pathway, especially BRCA1/2 mutated tumors. However, treatment is limited to specific sub-groups of patients and resistance can occur, limiting the use of PARPi. Poly(ADP-ribose) glycohydrolase (PARG) reverses the action of PARP enzymes, hydrolysing the ribose-ribose bonds present in poly(ADP-ribose). Like PARPs, PARG is involved in DNA replication and repair and PARG depleted/inhibited cells show increased sensitivity to DNA damaging agents. They also display an accumulation of perturbed replication intermediates which can lead to synthetic lethality in certain contexts. In addition, PARG is thought to play an important role in preventing the accumulation of cytoplasmic PAR and therefore parthanatos, a caspase-independent PAR-mediated type of cell death. In contrast to PARP, the therapeutic potential of PARG has been largely ignored. However, several recent papers have demonstrated the exciting possibilities that inhibitors of this enzyme may have for cancer treatment, both as single agents and in combination with cytotoxic drugs and radiotherapy. This article discusses what is known about the functions of PARP and PARG and the potential future implications of pharmacological inhibition in anti-cancer therapy.

## Introduction

**FIGURE 1:**
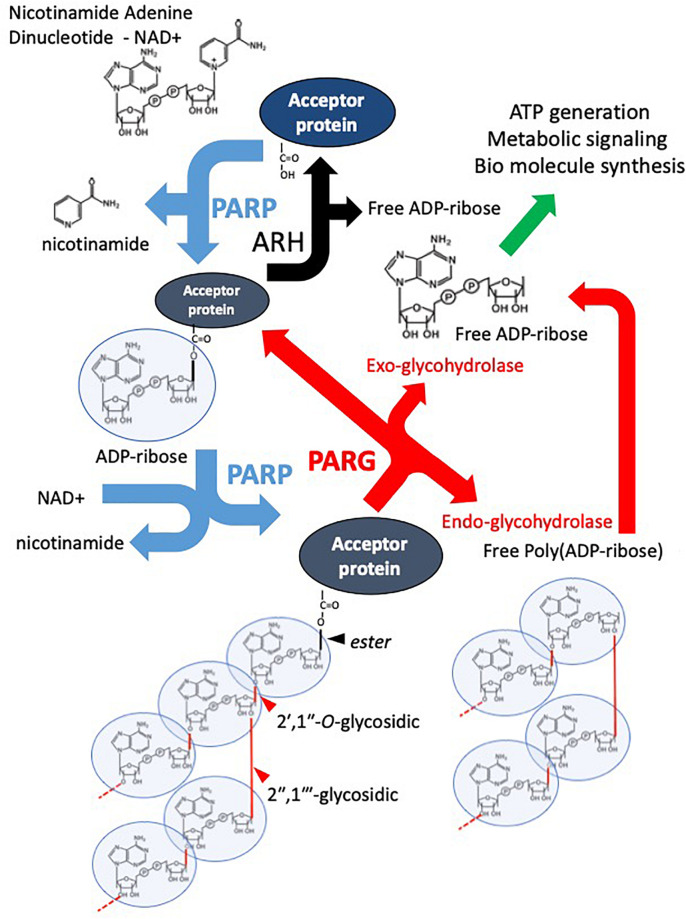
Overview of the PAR Cycle. PARP family enzymes use NAD+ as a substrate to catalyze the transfer of an ADP-ribose moiety to a receptive carboxyl group on an aspartate, glutamate, asparagine, arginine, or lysine residue of an acceptor protein. Nicotinamide is cleaved off in the reaction. The mono-ADP-ribosylated protein is referred to as being MARylated. PARPs 1-5 are also capable of catalyzing the addition of ADP-ribose to distal ADP-ribose residues forming a polymer of ADP-ribose – PAR, through 2′–1″ ribose-ribose glycosidic and 2″–1″′ ribose-ribose glycosidic bonds. PARG is an endo- and exoglycosidase, it catalyses PAR releasing free poly(ADP-ribose) and ADP-ribose. However, PARG cannot remove the most proximal ADP-ribose. This is removed by other ADP-ribose hydrolases (ARH). The resulting free ADP-ribose is catalyzed by ADP-ribose pyrophosphohydrolases such as the NUDIX family producing ribose-5-phosphate (R5P) and Adenine-mono-phosphate (AMP). They are used in the production of biomolecules and metabolism.

Poly(ADP-ribose) polymerases (PARPs) are a superfamily of multi-domain proteins each possessing a highly conserved (ADP-ribosyl)transferase (ART) domain that catalyzes the cleavage of nicotinamide adenine dinucleotide (NAD+) into nicotinamide and ADP-ribose (Chambon et al., 1963, 1966; Nishizuka et al., 1967; Sugimura et al., 1967; Hayaishi and Ueda, 1977; Okayama et al., 1977). The ADP-ribose unit is then transferred to an acceptor protein, or itself, on specific amino acid residues (glutamate, lysine, arginine, serine, and aspartate, and also more recently reported cystine, threonine, histidine, tyrosine, and phospho-serine (through the phosphate) (Vyas et al., 2014; Hendriks et al., 2019) (Figure 1). This transfer can occur in a monomeric or polymeric (linear or branched chain) manner depending on the particular PARP enzyme (Miwa and Sugimura, 1971; Miwa et al., 1979; Rolli et al., 1997; Ruf et al., 1998). The ADP-ribose moieties are generally considered to be added to the most distal ADP-ribose terminus (Taniguchi, 1987; Alvarez-Gonzalez, 1988), however, other models have been suggested (Ikejima et al., 1987). Poly(ADP-ribose) glycohydrolase (PARG) and other mono(ADP-ribose) (MAR) and poly(ADP-ribose) (PAR) erasers such as ADP-ribose hydrolases (ARHs), macrodomain-containing ADP-ribose erasers, and ADP-ribosyl lyase are responsible for the rapid removal of ADP-ribose moieties from modified proteins, thus recycling NAD+ back into the cellular system (Figure 1). The primary focus of this review is PARG, however, to facilitate understanding, it is important to place it in the context of PARP activity, function and therapeutic inhibition.

## The Structure and Enzymatic Activity of the PARP (ARTD) Family

The PARP family consists of at least 17 members, classified by domain structure or activity. PARPs1-5b share a conserved His-Tyr-Glu (H-Y-E triad) within the NAD+ binding pocket of the ART domain (Otto et al., 2005). This triad is predicative of PARylation capabilities. Of these enzymes PARP1 was the first to be discovered (Chambon et al., 1963, 1966; Nishizuka et al., 1967; Sugimura et al., 1967) and is the most intensely researched. PARP1 is classified as a nuclear DNA activated PARylator, capable of adding multiple ADP-ribose residues to a single acceptor. These PAR units have been reported up to hundreds of residues in size. Furthermore, PARP1 is capable of promoting branching within a growing PAR chain (Miwa and Sugimura, 1971; Miwa et al., 1979; Rolli et al., 1997; Ruf et al., 1998). PARP2 is considered to have similar activity to PARP1 (Miwa et al., 1979; Rolli et al., 1997; Ruf et al., 1998). Both PARP1 and 2 are capable of catalyzing multiple activities from the ART domain, i.e., the initial transfer of ADP-ribose to an acceptor protein, elongating this chain further by catalyzing a 2′–1″ ribose-ribose glycosidic bond and instigating branching by catalyzing a 2″–1″′ ribose-ribose bond (Figure 1). Both PARP1 and PARP2 are activated by DNA damage, with different lesions reported to activate activity to varying degrees (Benjamin and Gill, 1980; Amé et al., 1999; Eustermann et al., 2015). PARP3 is also activated by DNA damage, however, despite containing the H-Y-E triad, there is conflicting evidence regarding its MARylation and PARylation activity. Initially, PARP3 was considered only capable of MARylation, however, it is now known to PARylate NuMa (Boehler et al., 2011) and KU80 (Beck et al., 2014). In addition to modifying proteins, PARP3 can also MARylate and PARylate free double strand break (DSB) ends (Munnur and Ahel, 2017; Zarkovic et al., 2018). The ability to carry out mono- and poly(ADP-ribosyl)ation may be reflective of a more complex and nuanced role of PARP3 in different cellular processes. PARP4 is associated with the vault complex, a massive ribonucleoprotein complex of unclear function and has MARylation activity (Van Zon et al., 2003). PARP5a and 5b, also known as tankyrases 1 and 2 (TNK1 and TNK2), are not dependent on DNA damage for catalytic activation and are located more widely throughout the cell (Cook et al., 2002). They are also capable of PARylation activity, typically only producing oligomers up to 20 ADP-ribose residues in length (Rippmann et al., 2002). The significance of PAR length and size is not fully understood. The rest of the family (PARP6-17) are considered MARylaters (Bai, 2015), with PARP9 only showing activity when complexed to DTX3L (Yang et al., 2017). However, PARP13 does not possess catalytic activity (Vyas et al., 2014). The PARP name is therefore a misnomer. The family’s expansion and previous nomenclature inaccuracies/inconsistencies has resulted in a new nomenclature proposal (Hottiger et al., 2010). This nomenclature revolves around the enzymatic reaction (ART) and enzymatic structural markers (diphtheria-like, in reference to the presence of the H-Y-E triad motif in secreted ADP-ribosylating pathogenic diphtheria toxins by *Corynebacterium diphtheriae*), hence the name ARTD. However, for clarity and consistency with the literature the PARP nomenclature will be used here. As the focus of this review is PARG which reverses PARylation and not MARylation (Slade et al., 2011), the function of the PARP enzymes capable of PARylation will be briefly discussed.

## The Functions of Parylating Enzymes

### Poly(ADP-Ribose) Polymerase 1, 2, and 3

**FIGURE 2:**
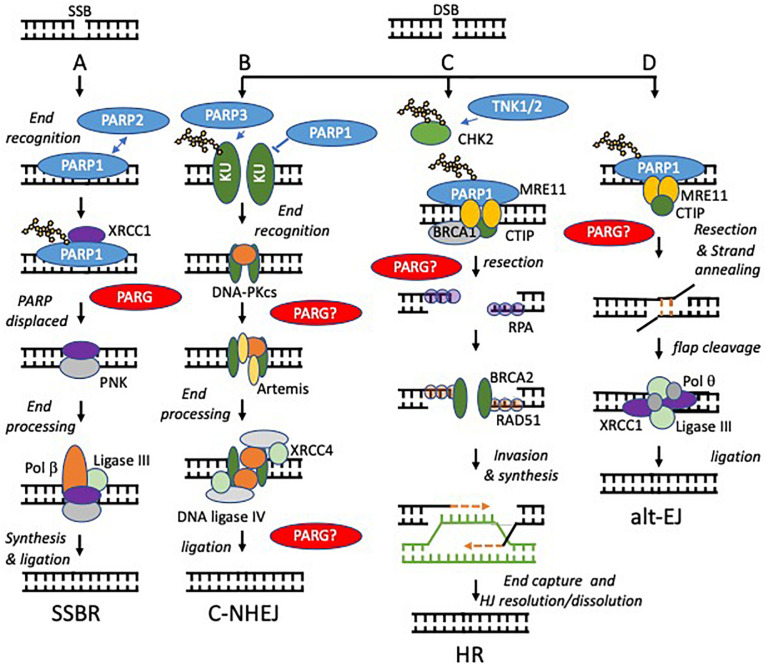
Models of PARP function at single and double strand breaks. Potential sites where PARG function is required to reverse PARylation to facilitate downstream processing are indicated by PARG?, proven roles indicated by PARG. **(A)** SSBs, arising directly from damage to the DNA backbone or as intermediates in BER, are bound by PARP1 or PARP2. DNA binding activates PARP causing automodification. Automodified PARP recruits XRCC1 to the SSB. PARP is displaced. Damaged 5’- or 3’-termini are processed into 5’-phosphate and 3’-hydroxyl groups by APE1 or PNK. Polβ then performs gap filling followed by ligation by Lig3α. **(B,C)** PARP activity regulates the relative contribution that non-homologous end-joining (NHEJ) and homologous recombination (HR) make to repair of DNA double strand breaks (DSBs). NHEJ begins with binding of the DNA ends by the Ku70/80 heterodimer, which recruits DNA-PKcs. If the ends are not compatible, they are trimmed by nucleases, e.g., Artemis. The ligation complex XRCC4-DNA Ligase IV-XLF then seals the DSB. In HR, MRE11 resects the break to generate single stranded DNA (ssDNA), which is quickly coated and subsequently replaced by Rad51. The Rad51 nucleoprotein filaments mediate strand invasion of the homologous template. Synthesis of DNA using the sister chromatid is then followed by capture of the second end and holiday junction (HJ) resolution or dissolution leading to DSB repair. PARP1 competes with KU for binding at DSBs and promotes resection by MRE11 (a component of the MRN complex, therefore PARP1 activation favors HR. In contrast PARP3 PARylates KU70/80and limits DNA end resection favoring NHEJ. TNK1/2 PARylates CHK2 to promote HR. **(D)** PARP1 is required to recruit MRE11 to regions of DNA micro-homology in order to initiate alternative end-joining (alt-EJ), following limited resection and strand annealing, the DNA flaps are cleaved, finally Pol q and the ligation complex XRCC1-ligase III seal the gap.

**FIGURE 3:**
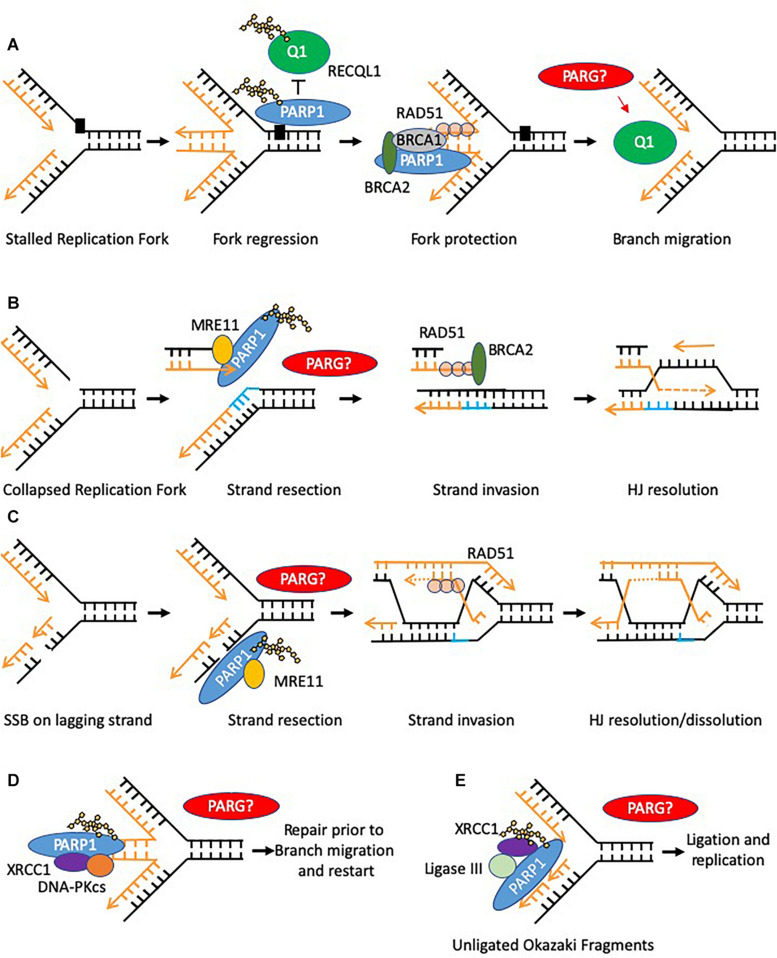
Models of PARP function at replication forks. Potential sites where PARG function is required to reverse PARylation to facilitate downstream processing are indicated by PARG? **(A)** PARP is activated at stalled replication forks and poly(ADP-ribosyl)ates RECQ1 thus constraining its branch migration activity. In addition PARP binds and protects stalled forks form excessive nuclease activity. **(B,C)** PARP activation occurs at collapsed replication forks or gaps in the lagging strand post-replication, in each case promoting MRE11 to resect the leading strand template allowing for HR–mediated fork recovery. **(D)** PARP can recruit XRCC1 and DNA-PK to stalled reversed replication forks. However, only a small defect in the restart of stalled forks is seen when PARP activity is absent, suggesting this function is to mediate repair of a subset of stalled forks, perhaps where small lesions impede re-start of the forks **(E)** PARP can bind to and is activated at unligated Okazaki fragments during DNA replication, promoting recruitment of XRCC1 and ligase III thus facilitating repair and continued replication.

PARP1, 2, and 3 contain DNA binding domains that facilitate their interaction with DNA and enable them to PARylate target proteins, perform auto-modification and even modify free DNA ends (Vyas et al., 2014). PARylation has been observed to have multiple context dependent consequences. One is that the modified proteins operate as a recruitment platform for other proteins. For example, efficient resolution of single strand breaks (SSBs) is facilitated by XRCC1 (Whitehouse et al., 2001), the recruitment of which is in turn enhanced by interaction with DNA bound automodified PARP1 and 2 at sites of damage (Masson et al., 1998; Okano et al., 2003; Fisher et al., 2007; Hanzlikova et al., 2016). Additional consequences of PARylation on an acceptor protein include DNA/RNA dissociation due to the strong negative charge of PAR (Satoh and Lindahl, 1992), and acceptor protein topography changes influencing protein-protein interactions or modulating acceptor protein catalytic activity (Muthurajan et al., 2014; Fischbach et al., 2018; Zhen and Yu, 2018; Yang et al., 2020). Finally PARPs are reported to MARylate and PARylate DNA/RNA ends directly, although the functional consequence of this is not clear (Audebert et al., 2004; Wang et al., 2006; Fisher et al., 2007; Gao et al., 2007; Haince et al., 2008; Talhaoui et al., 2016; Munnur and Ahel, 2017; Zarkovic et al., 2018; Munnur et al., 2019). Through these modes of action, PARylation by PARP1 and/or 2 has been reported to influence multiple pathways of DNA repair including, single strand break repair (SSBR) (Fisher et al., 2007; Hanzlikova et al., 2016), homologous recombination (HR) (Haince et al., 2008), non-homologous end-joining (NHEJ) (Wang et al., 2006), and alternative non-homologous end-joining (alt-EJ) (Audebert et al., 2004) (Figure 2). They are also required for DNA replication fork stability under conditions of replication stress (Figure 3) (Sugimura et al., 2008; Bryant et al., 2009; Petermann et al., 2010; Min et al., 2013; Ying et al., 2016). PARP1 is thought to account for approximately 90% of the cell’s PARylation activity. However, PARylation still occurs in PARP1 deficient cells (Shieh et al., 1998; Beneke et al., 2000; Dantzer et al., 2000; Masutani et al., 2000) suggesting that PARP1 and 2 have overlapping roles and that PARP2 may be able to partially compensate for PARP1 loss. For example, both are capable of recruiting XRCC1 to chromatin for SSBR (Fisher et al., 2007; Hanzlikova et al., 2016), promoting nucleolar transcription via a mutual interaction with B23 (Meder et al., 2005), possessing partially overlapping interactomes (Isabelle et al., 2010) and both engaging in replication fork stabilization (Ronson et al., 2018). Supportive of compensatory roles, co-deletion of PARP1 and PARP2 results in embryonic lethality (Menissier De Murcia, 2003), however, PARP1−/− mice exhibit increased spontaneous tumor incidence suggesting PARP2 cannot fully compensate for PARP1 loss (Shibata et al., 2009). In contrast to PARP1/PARP2 double knockout mice, PARP1/PARP3 knockout animals are viable suggesting a separate function for PARP3 (Boehler et al., 2011). However, this does not exclude a separate role for PARP3 in DNA repair, especially as the PAR activity of PARP3 is activated by different types of breaks to PARP1 (Langelier et al., 2014). In this regard PARP3 is implicated in DNA double strand break repair (DSBR) where it may work in concert with PARP1 to help coordinate repair by classical non-homologous end-joining (c-NHEJ) (Figure 2) (Boehler et al., 2011; Rulten et al., 2011; Fenton et al., 2013; Beck et al., 2014). Outside of DNA repair, nuclear PARPs have also been implicated in transcription, chromatin modification and cell death pathways (Yu et al., 2006; Szántó et al., 2012; Dantzer and Santoro, 2013; Muthurajan et al., 2014). Interestingly, the balance between auto- and trans-ribosylation is suggested to play a role in regulating PARP function, with trans-modification of histones limiting auto-modification of PARP1/2 in a HPF1 dependent fashion (Gibbs-Seymour et al., 2016; Palazzo et al., 2018; Suskiewicz et al., 2020).

In summary, PARP1-3 are all localized to the nucleus, each is activated by free DNA ends and is implicated in many cellular processes to varying degrees. One of the key ways in which PARP1-3 function is via the addition of a large post-translational modification onto themselves and other proteins. PARG functions to reverse this modification and thus absence or inhibition of PARG is likely to alter the kinetics of dePARylation, leaving proteins inappropriately modified. This is likely to have a similarly complex, overlapping and, perhaps also a unique set of consequences as the lack of PARylation induced by PARPi.

For more comprehensive reviews of PARP1-3 biology (see Bai, 2015; Gupte et al., 2017; Ray Chaudhuri and Nussenzweig, 2017).

### The Tankyrases – PARP 5a and 5b

Tankyrase 1 (TNK1) and tankyrase 2 (TNK2) (also known as PARP5a and 5b) are a distinct subgroup of the PARPs found in the nucleus, golgi apparatus, cytoplasm and at telomeres [reviewed in Kim (2018)]. They are capable of homo and hetero dimerization via their sterile alpha module (SAM) domain and can be activated and auto-modified in a DNA independent manner. Both produce oligomers of ADP-ribose typically up to 20 units in length (Smith, 1998; Rippmann et al., 2002; De Rycker and Price, 2004). TNK1 and TNK2 are considered scaffold proteins; they contain 24 ankyrin domains, divided into 5 ankyrin (ANK) repeat clusters (ARC) that individually are capable of binding to tankyrase interacting proteins (TIPs). Many of the TIPs are also substrates of TNK1 and TNK2. TIPs are a diverse group of proteins spanning numerous cellular processes. Consistent with this, tankyrases have reported roles in DNA repair, including stabilizing and PARylating DNA-PK and checkpoint protein 2 (CHK2) (Dregalla et al., 2010; Nagy et al., 2016; Okamoto et al., 2018), telomerase-dependent telomere length maintenance (Smith, 1998; Smith and De Lange, 2000), spindle assembly, centrosome maturation, resolution of sister telomeres during mitosis (Dynek and Smith, 2004; Chang et al., 2005, 2009; Canudas et al., 2007; Ozaki et al., 2012), Wnt, Notch, AKT and YAP signal transduction pathways (Huang et al., 2009; Li et al., 2015; Mariotti et al., 2016, 2017; Troilo et al., 2016; Yang et al., 2016; Jia et al., 2017), and regulation of glucose metabolism (Chi and Lodish, 2000; Yeh et al., 2009; Zhong et al., 2016). Most tankyrase functions are considered to be mediated via PARylation, altering protein:protein interactions. Often but not exclusively, tankyrase-mediated PARylation is recognized and ubiquitinated by the E3 ligase RNFL146 (Zhang et al., 2011), this targets substrates for proteasomal degradation and thus regulation is commonly via alteration of protein stability. However, evidence is also emerging that tankyrases can have catalytic independent functions (Pollock et al., 2019). As tankyrase biology is relatively less well understood than that of PARP1-3, it is likely that further, unknown tankyrase functions exist. The ankyrin domains within TNK1 and TNK2 share 83% homology and it is thought that many of the TIPs overlap. Where investigated this has been confirmed to be the case; indeed double tankyrase knockout is embryonic lethal whereas single tankyrase knockout mice have different yet mild phenotypes (Chiang et al., 2006, 2008; Hsiao et al., 2006). This implies distinct interacting partners and functions exist. It is suggested that PARG inhibition will prevent removal of tankyrase catalyzed-PAR, thus inhibition of PARG is likely to impinge on many tankyrase regulated pathways (Gravells et al., 2018). However, this activity for PARG has not been formally demonstrated.

## PARP Inhibition (PARPi)

### PARP Inhibitors

Inhibition of PARPs can be mediated by small molecules containing nicotinamide/benzamide pharmacores that dock into the NAD+ pocket within the ART domain. Here they act as competitive inhibitors of NAD+, preventing ADP-ribose transferase activity (Preiss et al., 1971; Durkacz et al., 1980; Purnell and Whish, 1980; Marsischky et al., 1995; Canan Koch et al., 2002; Skalitzky et al., 2003; Calabrese et al., 2004; Ferraris, 2010). Conservation within the catalytic pocket makes specific inhibition of PARPs challenging and despite multiple sophisticated drug discovery programs even many of the latest and clinically relevant PARP1/2 inhibitors are not completely selective, with inhibition of PARP3 and tankyrases being the most common targets of cross-reactivities (Carney et al., 2018). The cytotoxicity of PARP inhibition is considerably greater than loss of PARP; an explanation for this is that catalytically inactive PARP can become “trapped” on DNA (Satoh and Lindahl, 1992; Murai et al., 2012). As inhibitors with similar inhibitory activities display different trapping abilities (Murai et al., 2014), it has been proposed that interactions between an inhibitor and the PARP NAD binding domain can lead to allosteric interactions within the DNA binding domain of PARP, tightening the interaction between PARP and DNA (Langelier et al., 2018). Furthermore, once bound to DNA in the presence of an inhibitor, PARP is “trapped” because inhibition of catalytic activity prevents the auto-ribosylation that promotes PARP-DNA dissociation (Shen et al., 2015; Langelier et al., 2018).

The therapeutic contribution of relative trapping potencies and specificity of each inhibitor against PARP1/2/3/5a and 5b proteins is yet to be fully determined. Whether PARP1-3 and TNK1/2 are trapped on DNA when PARG is inhibited is yet to be fully determined but will likely influence the potency of PARGi.

### PARPi Sensitize to DNA Damaging Agents

The characterization of PARP1’s involvement in DNA repair quickly led to the realization that inhibitors may potentiate the standard treatment modalities employed in oncology, such as DNA damaging agents, particularly temozolomide, and radiotherapy (Durkacz et al., 1980; Sakamoto et al., 1983; Arundel-Suto et al., 1991; Suto et al., 1991; Banasik et al., 1992; Satoh and Lindahl, 1992; Griffin et al., 1995; Bowman et al., 1998; Calabrese et al., 2004). Drug discovery pipelines eventually produced more clinically viable inhibitors with greater potency, specificity, drug solubility and bioavailability (Curtin, 2014) and the first clinical trial was a combination of the PARPi rucaparib and temozolomide (Plummer et al., 2008). There are currently many clinical trials being undertaken with different inhibitors of PARP namely olaparib, rucaparib, veliparib, niraparib, and talazoparib, in a range of cancers (De Bono et al., 2017; Jiang et al., 2019; Tuli et al., 2019), and their capacity to act as chemo/radio-sensitizers is well documented. A comprehensive review of PARP inhibitor development for cancer including stratification of patients, biomarker identification and combination strategies can be found here (Davar et al., 2012; Mateo et al., 2019).

### PARPi Is Synthetically Lethal With Defects in Homologous Recombination

In cells with loss of function mutations associated with breast cancer susceptibility genes BRCA1 or 2, inhibition of PARP was demonstrated to be synthetically lethal (Bryant et al., 2005; Farmer et al., 2005). Bryant et al. (2005) extended their study to include other genes involved in HR suggesting the mechanism stems from a relationship between PARP and the HR pathway. This observation has resulted in a paradigm shift and many other synthetically lethal relationships including others with PARP, have been described (Turner et al., 2008). The mechanism of action underpinning the sensitivity of BRCA mutated cells to PARP1 inhibition is thought to be multifactorial (Dziadkowiec et al., 2016). PARylation by PARP1 and 2 is involved in SSBR, the protection of replication forks and fork restart (Figures 2, 3). Therefore, catalytic inhibition can lead to an increase in unresolved DNA lesions and stalled forks that can collapse and then translate to DSBs in DNA. PARP inhibitors can also cause PARP to be “trapped” on DNA (Murai et al., 2012). This trapped protein is itself a form of DNA lesion, thus when trapped PARPs collide with replication forks, this can stall replication forks and may produce DSBs, which is likely to compound the potency of those PARPi with greater trapping ability. HR is required to resolve DSBs, and many HR proteins are also required for fork protection/restart during replication stress (Slack et al., 2006; Payen et al., 2008; Mizuno et al., 2009; Ait Saada et al., 2018). PARPi therefore increase the degree to which cancer cells are reliant on core and regulatory HR factors. Deficiency in HR related proteins such as BRCA1/2 then when combined with a PARPi result in unrepaired lesions which can lead to apoptosis and/or mitotic defects and death via mitotic catastrophe.

### Limitations of PARPi

PARP inhibition is providing positive results in the clinic. However, like any advancement made in oncology, there are limitations. Firstly, PARP trapping by inhibitors compounds the catalytic inhibition of the PARP enzyme, increasing their potency. However, different PARP inhibitors exert the PARP trapping effect to varying degrees and it can result in off-target PARP trapping on the DNA of healthy tissue (Hopkins et al., 2019). How to best optimize this for therapeutic gain is yet to be fully determined. Secondly, various PARP inhibitors have differential affinities for other PARP’s, posing a challenge for therapeutic specificity. Finally, there is the emergence of PARP inhibitor resistance within the clinic. Potentially underlying mechanisms have been identified using pre-clinical models. These mechanisms briefly include increased expression of drug efflux proteins (Rottenberg et al., 2008; Vaidyanathan et al., 2016), loss of PARP trapping (Gogola et al., 2018; Pettitt et al., 2018), restoration of HR (Bouwman et al., 2010; Bunting et al., 2010; Jaspers et al., 2013; Ter Brugge et al., 2016; Goodall et al., 2017; Dev et al., 2018; He et al., 2018; Mirman et al., 2018; Noordermeer et al., 2018) and replication fork stabilization (Chaudhuri et al., 2016; Taglialatela et al., 2017; Murai et al., 2018) (Table 1). Given these limitations, additional drug targets that are effective against BRCA proficient and deficient tumors or indeed, even against PARP inhibitor resistant tumors, are needed. One promising target of interest is poly(ADP-ribose) glycohydrolase (PARG).

**TABLE 1:** PARP inhibitor resistance mechanisms.

**Resistance mechanism**	**Cause of resistance**	**Pre-clinical and clinical observations**	**References**
HR restoration	BRAC1/2 Reversion restoring HR	Mutations in patient tumors and PDX models treated with PARPi, reversion mutations in BRCA1 and BRCA2 occur frequently in patients with PARPi-resistant cancers	Ter Brugge et al., 2016; Goodall et al., 2017
	Demethylation of hypermethylated BRCA1 promoter	PDX models treated with PARPi	Ter Brugge et al., 2016
	Loss of 53BP1	Low expression and mutations in BRCA1 deficient PDX models	Bouwman et al., 2010; Bunting et al., 2010
	Loss of Shieldin factors	Low expression and Mutations in BRCA1 deficient PDX model	Dev et al., 2018; Noordermeer et al., 2018
	Loss of CTC/Pola	*In vitro* observations that phenocopy 53BP1 loss	Mirman et al., 2018
	Loss of DYNLL1/ATMIN	*In vitro* reports that partially phenocopy 53BP1 loss	He et al., 2018
Stalled fork stabilization	Loss of PTIP, SLFN11 and SMARCAL1	*In vitro* reports of loss inducing PARPi resistance in BRCA1/2 deficient cells	Chaudhuri et al., 2016; Taglialatela et al., 2017; Murai et al., 2018
	Loss of EZH2	*In vitro* reports of loss inducing PARPi resistance in BRCA2 deficient cells	Rondinelli et al., 2017
Loss of PARP trapping	Mutation in PARP	*In vitro* reports of mutations in PARP that induce resistance in BRCA2 deficient cells, PARP1 mutation (R591C) which prevents trapping found in a de novo PARPi resistant patient tumor	Pettitt et al., 2018
	Loss of PARG	*In vitro* PARG depletion can cause PARPi resistance in BRCA2−/− background	Gogola et al., 2018
Increased drug efflux	ABC transporter upregulation	PARPi resistance *in vitro* and in mouse models	Rottenberg et al., 2008; Jaspers et al., 2013; Vaidyanathan et al., 2016

## PARG – the Primary Mediator of PAR Catabolism

### Enzymology and Catalysis

Poly (ADP-ribose) glycohydrolase is the primary hydrolase involved in the degradation of PAR (Figure 1) (Miwa and Sugimura, 1971; Miwa et al., 1974; Lin et al., 1997).

PARG possesses both endo-glycohydrolase and exo-glycohydrolase activity, preferentially performing the latter by binding to the two most distal ADP-ribose residues within the PAR chain (Brochu et al., 1994; Barkauskaite et al., 2013). These different modes of catalysis produce free PAR and mono ADP-ribose moieties, respectively. The free mono ADP-ribose is then metabolized into AMP and ribose 5′ phosphate by ADP-ribose pyrophosphohydrolases such as the NUDIX family (Mildvan et al., 2005; McLennan, 2006; Palazzo et al., 2015). AMP is utilized in ATP reformation and different metabolic and cell signaling pathways (Rodríguez-Vargas et al., 2019) while ribose 5′ phosphate is a precursor to many biomolecules including DNA, RNA and ATP (Kowalik et al., 2017). Endo-glycohydrolase activity is considered to occur primarily during hyper-PARP activation, the resulting free PAR chains produced are then implicated in apoptosis acting as a death signal (Andrabi et al., 2008). PARG itself specifically catalyzes the hydrolysis of α(1″–2′) or α(1″′–2″) glycosidic linkages (Figure 1). Although PARG is the primary PAR hydrolase, some redundancy with the less efficient ADP-ribosylhydrolase 3 (ARH3, also called ADPRHL2) has been reported (Mueller-Dieckmann et al., 2006; Oka et al., 2006; Fontana et al., 2017). Interestingly, other studies indicate a unique function for ARH3 in degradation of mitochondrial PAR (Niere et al., 2012). A more comprehensive review on other PAR and MAR hydrolases can be found in O’Sullivan et al. (2019).

### The PARG Isoforms – Subcellular Localization and Domain Architecture

**FIGURE 4:**
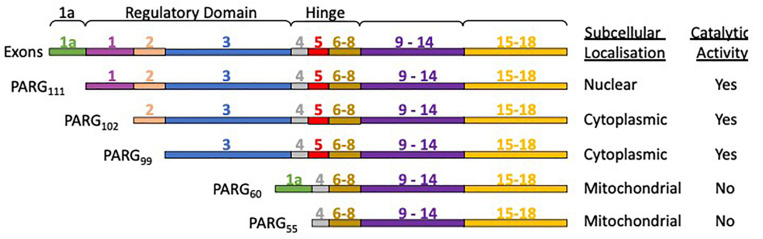
PARG isoforms are the product of a single gene and alternative splicing. PARG111 contains all the exons, PARG 102 lacks exon 1, and PARG99 lacks exon 1 and 2. PARG60 has exons 1a, 4 and 6-18. PARG55 is the same but lacks exon 1a. PARG60 and 55 lack exon 5 rendering them catalytically inactive.

The human PARG gene is located at a single chromosomal locus 10q11.23-21 (Shimokawa et al., 1998; Ame et al., 1999). However, the PARG gene transcription product is subject to alternative splicing, producing different PARG isoforms (Figure 4) with distinct subcellular localization (Meyer-Ficca et al., 2004). PARG111 is the largest isoform and contains four domains: an intrinsically disordered regulatory region, a hinge domain, the PARG catalytic domain and a macrodomain. It is the primary nuclear PARG and has been reported to translocate to the cytoplasm. PARG 102 and 99 lack part of the N-terminal domain and possess a greater degree of whole cell activity. They have a cytoplasmic and perinuclear distribution and have been observed to translocate to the nucleus (Winstall et al., 1999), particularly during genotoxic insult (Haince et al., 2006). The details of how the shuttling of different isoforms fully contributes to PAR metabolism and its significance is yet to be elucidated. Mitochondrial PARG55 and PARG60 lack catalytic activity (Meyer et al., 2007; Whatcott et al., 2009; Niere et al., 2012). Functions have yet to be attributed to them. We hypothesize they may have a PAR binding/regulatory role that is independent of catalytic activity. Deletion of the PARG gene results in embryonic lethality in mice making study of PARG by complete genetic deletion difficult (Koh et al., 2004). Homozygous deletion of PARG in exons 2 and 3 results in a deletion of the PARG110 isoform (equivalent to human 111 isoform) and is tolerated in animals (Cortes et al., 2004). The manner in which the rest of the exons are distributed across isoforms means other genetic manipulations, selectively removing other isoforms, have not been possible.

## PARG Inhibitors

Most PARG studies have until recently relied on hypomorphic variants of PARG or RNAi-mediated depletion to examine function. Development of PARG inhibitors (PARGi) has been late compared to PARPi. Early inhibitors were restricted to a large naturally occurring polyphenol, gallotannin, or rhodanine-based inhibitors. However, increasingly cell permeable and specific PARG inhibitors have recently been developed (Table 2). These cluster into three types, quinazolinedione-type (PDD00017273), naphthalen-type (COH34) and thioxanthine/methylxanthine derivatives (JA2-4 and JA2131). All compete for PAR in the PARG active site (James et al., 2016; Chen and Yu, 2019; Houl et al., 2019). These inhibitors have helped elucidate function. Regardless, an analogous problem remains that all the inhibitors developed to date, work by inhibiting all catalytically active PARG isoforms. The significance of inhibiting some or all of the PARG isoforms has yet to be explored.

**TABLE 2:** PARG inhibitors.

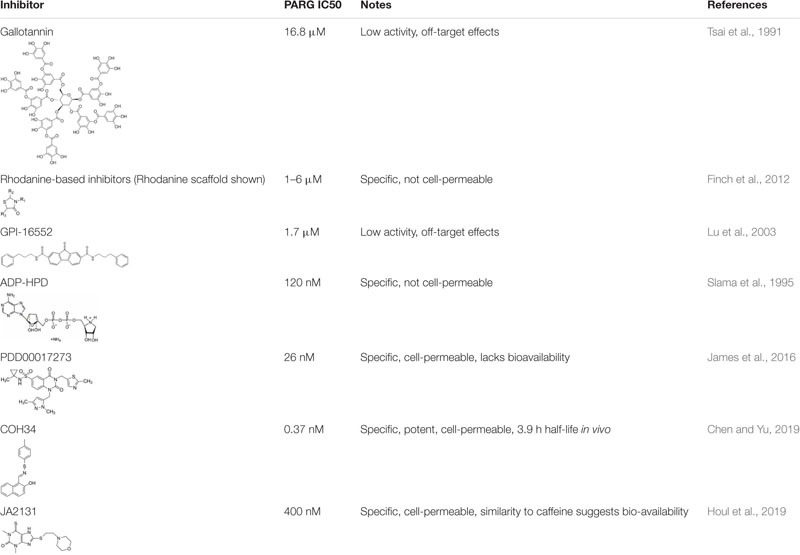

## PARG Functions in DNA Repair

The consensus opinion in PAR biology is that PARP and PARG co-operate to facilitate downstream cellular processes. Like PARP, PARG loss and inhibition sensitizes cells to DNA damaging agents, and it is likely that the function of PARG is to work with various PARP proteins to facilitate an appropriate and timely DNA repair. However, distinct functions should not be ruled out.

### Single Strand Break Repair

**FIGURE 5:**
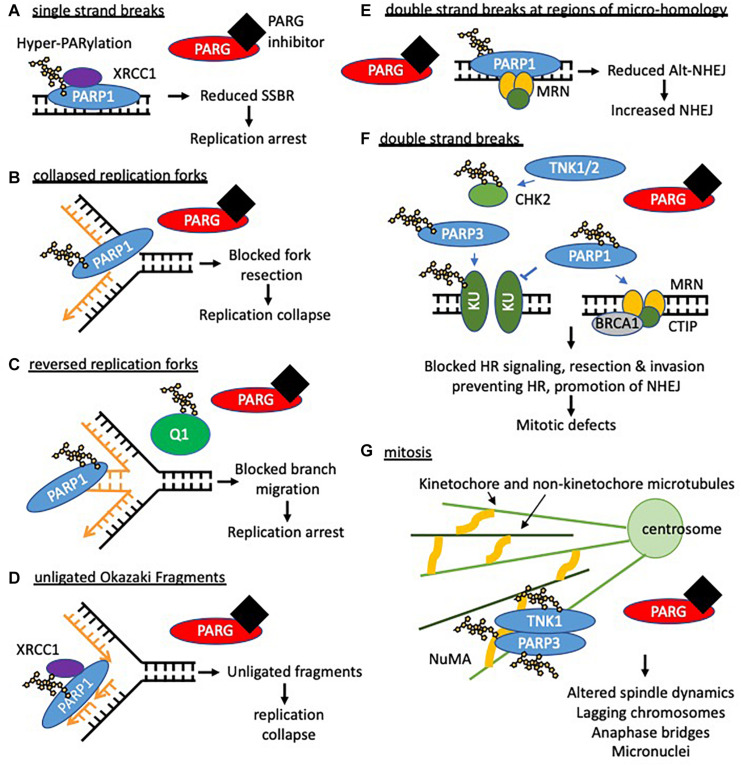
Potential consequences of PARG inhibition (compared to PARP inhibition). Loss of dePARylation leads to increased PAR levels on PARP and other acceptor proteins. This may prevent dissociation of PAR binding proteins preventing downstream processing and/or trap PARP on DNA forming further lesions perturbing replication and leading to S/G2 phase arrest. If instability at replication forks persists or in the presence of DSBs cells will undergo apoptosis or mitotic catastrophe. In this context both synthetic lethality with compensatory pathways needed to maintain replication integrity (HR and replication complex proteins) and radio- and chemosensitization can occur. In addition it is likely that prolonged PARylation of other acceptor proteins modifies their function and impacts on cell behavior including survival. **(A)** When PARG is inhibited hyper-PARylated PARP1/2 and XRCC1 accumulate at DNA single strand breaks such that single strand break repair (SSBR) cannot proceed. In addition trapped PARP will form a lesion, which is likely to perturb replication. PARP inhibition traps unmodified PARP at SSBs which slows repair and also collapses/stalls replication. **(B)** Activated PARP promotes collapsed fork restart, it is possible that reversal of PARylation on PARP is required for fork restart (by homologous recombination (HR)) such that PARG inhibition prevents restart. PARP inhibition also prevents fork restart at collapsed forks. **(C)** PARG inhibition leads to accumulation of reversed forks following replication stress likely because RECQ1 (Q1) remains PARylated and thus cannot promote branch migration for replication restart. This is in contrast to PARP inhibition where reduced levels of reversed forks are seen. **(D)** PARG inhibition leads to an increase in unligated Okazaki fragments. Analogous to SSBR it is likely that hyper-PARylated PARP accumulates XRCC1 but prevents downstream ligation of okazaki fragments leading to replication fork instability. PARP inhibition slows ligation of fragments. **(E,F)** PARG inhibition combined with DNA damage induced double strand breaks (DSB) is likely to lead to trapped hyper-PARylated PARP on DNA repair substrate intermediates and increased PARylation of KU80 and CHK2, altering downstream repair of DNA and resulting in mitotic defects. PARP1/2 inhibition causes increased NHEJ. **(G)** PARG inhibition leads to mitotic defects including fragmentation and amplification of centrosomes, multipolar spindles, chromosomal misalignment and aberrant segregation of chromosomes. This is likely due to unrepaired DNA damage persisting into mitosis (see above) and lack of reversal of tankyrase (TNK1) and PARP3 dependent-PARylation during mitosis. E.g., during mitosis PARP3 stimulates TNK activity increasing auto-ribosylation and ribosylation of NuMA to coordinate spindle dynamics. It is possible that in the absence of dePARylation mitosis cannot progress, and that in the presence of exogenous DNA damage or inhibition of checkpoint signaling cells die of mitotic catastrophe.

PARG has been implicated in SSBR by both potentiating the number of SSBs (Zhou et al., 2010) and reducing repair kinetics (Fisher et al., 2007; Ame et al., 2009; Min et al., 2010; Chen and Yu, 2019). PARG depletion reduced the repair kinetics of hydrogen peroxide induced SSBs. Concomitant depletion of PARP1 did not further reduce the repair kinetics (Fisher et al., 2007). This suggests PARP1 and PARG act in the same pathway to promote SSBR. Following the induction of SSBs, PARP1/2 senses the damage, auto-PARylates itself and recruits XRCC1 to the damaged site (Figure 2). XRCC1 then recruits the rest of the SSBR factors finalizing the repair of the lesion. It is suggested that removal of PAR is required to allow efficient repair (Figure 5). In support of this, auto-modified PARP1 accumulated and persisted at sites of SSBs when PARG was inhibited or depleted (Wei et al., 2013; Gogola et al., 2018). Furthermore, XRCC1 was retained at these same sites for longer (Fisher et al., 2007; Wei et al., 2013; Chen and Yu, 2019). Interestingly, mouse cells deficient in exon 2 and 3 of PARG resulted in fewer XRCC1 foci formation in response to methylnitronitrosoguanidine (MNNG) treatment (Gao et al., 2007). This suggests there may be a relationship between the nuclear PARG isoform and XRCC1. In support of this, PAR removal has been demonstrated to facilitate XRCC1 translocation from PAR directly to the SSB (Wei et al., 2013) and if PARG activity is compromised then it may reduce the efficiency at which this can occur. This may partially explain the reduced repair kinetics. It is also possible that persistence of PARP1 and XRCC1 at damage sites limits the availability of these molecules for repair at other sites. It is likely in the absence of dePARylation, PARP1 complexes bound to SSBs serve as a lesion themselves. Furthermore, it is likely that these lesions could act to form a barrier to replication and thus result in stalled or collapsed replication forks (Figure 5). Support for this comes from PARGi treated cells where increased levels of DNA damage were dependent on replication (Fathers et al., 2012). Furthermore, PARG deficient −/− embryonic stem cells and PARG depleted pancreatic cancer cells exhibited S-phase arrest and increased DNA damage when treated with the alkylating agent MMS (Shirai et al., 2013b). As PARG is recruited to PAR via its macrodomain, it is not clear whether when PARG is inhibited PARG protein remains bound to PAR.

### Double Strand Break Repair, Replication, and Replication Fork Stability

The relationship between PARG and DSBR is poorly understood but given the key role various PARPs play in the regulation of DSBR, it is likely that reversal of PARP1-3 activity during HR, alt-EJ and/or NHEJ by PARG is important for accurate repair (Figure 5). Indeed, PARG depletion and inhibition have both been shown to slow the repair kinetics of radiation-induced DSBs with changes in both HR and NHEJ reported (Ame et al., 2009; Gravells et al., 2018; Chen and Yu, 2019). However, problems with replication can lead to forks collapsing into DSBs, and with PARPs playing a prominent role in maintaining fork stability, separating a role for PARG in DNA replication and a role in DSBR is difficult. Interpretation of experiments is further compounded by the finding that a marker of HR at DSBs, RAD51, can also be a signal for fork stabilization and HR mediated restart of replication forks (Bhattacharya et al., 2017), while a marker of DSBs 53BP-1 is also known to be involved in fork protection (Her et al., 2018).

The first study to suggest PARG is associated with replication showed that following hydroxyurea (HU)-induced replication stress, PARG110 deficient mouse cells display increased and prolonged RAD51 foci formation (Min et al., 2010). More direct evidence suggesting PARG111 has a role in DNA replication comes from its association with proliferating cell nuclear antigen (PCNA). GFP-tagged PARG111 co-localizes with PCNA throughout S-phase while the 102 and 99 PARG isoforms do not. Immunoprecipitation confirmed the N-terminal residues mediated an interaction (Mortusewicz et al., 2011). Kaufmann et al. (2017) later confirmed this N-terminal sequence was indeed important for replication foci association, however, the interaction was primarily mediated via the acetylation of a non-canonical PIP box in exon 3 of PARG (Kaufmann et al., 2017). This explains why co-immunoprecipitation of the PARG102/99 isoforms with PCNA was present although substantially reduced relative to PARG111 (Mortusewicz et al., 2011). We hypothesize that preventing PARG K409 acetylation or disrupting the protein-protein interaction with PCNA could be exploited to specifically target the nuclear PARG111 isoform. It is unclear whether the PARG/PCNA interaction is integral to replication in an unchallenged environment or whether it only has function during replication stress.

PARG depletion increased the levels of PAR following chronic but not transient HU-induced replication stress (Illuzzi et al., 2014). Increased PAR was associated with reduced levels of chromatin bound and phosphorylated RPA. We can therefore hypothesize that excessive PAR can prevent RPA binding to collapsed but not stalled replication forks. Further support for a function for PARG during replication stress comes from analyzing single replication forks using electron microscopy and DNA fiber assays (Ray Chaudhuri et al., 2015). In this study, PARG depletion slowed replication forks and increased the number of reversed replication forks (Ray Chaudhuri et al., 2015). There was also an increase in S-phase associated γH2AX staining, a strong ATM and ATR signal and an increase in chromatin binding of RAD51 and 53BP1. However, there were no detectable DSBs by pulsed field gel electrophoresis. The recruitment of these repair proteins in a context where there were no detectable DSBs suggests that their recruitment is to facilitate their replication associated functions. Thus, when PARG is compromised, there may be a reliance on replication fork protecting/restart factors. This may explain why PARPi resistant cells with partially restored HR activity due to 53BP1 mutations are sensitive to PARG inhibitors (Chen and Yu, 2019). Consistent with PARG depletion, PARG inhibition also slowed forks and increased fork stalling as shown by the DNA fiber assay, and led to increased γH2AX and RAD51 foci formation on chromatin (Gravells et al., 2017; Houl et al., 2019), adding further evidence for a function of PARG during replication.

Although reduced PARG function leads to perturbed replication, the precise function of PARG is not clear. PARP is reported to both protect transiently stalled replication forks from collapse and mediate collapsed fork restart (Bryant et al., 2009; Petermann et al., 2010; Ying et al., 2016). It is possible that as with SSBR, removal of PAR from PARP is required to facilitate the downstream steps of fork restart, although no direct evidence for this exists (Figures 3, 5). In addition, dePARylation of other proteins may be important. RECQ1 is a replication fork associated helicase involved in replication fork restart following fork reversal at sites of stalling (Popuri et al., 2012). PARP1 PARylates RECQ1 inhibiting its action and preventing premature fork restart and fork collapse (Ray Chaudhuri et al., 2012; Berti et al., 2013). RECQ1 and PARG depletion phenocopied each other to prevent fork restart in telomeric DNA suggesting a function for PARG in reversing RECQ1 inhibition (Margalef et al., 2018). It is not known whether PARG regulates RECQ1 activity in non-telomeric DNA. However, if it occurs, lack of PARG during DNA replication could lead to destabilization of reversed forks and an increased requirement for fork protection factors to prevent collapse (Figures 3, 5). This may partially explain why fork protection factors are recruited to chromatin in a PARG deficient background (Ray Chaudhuri et al., 2015). Further evidence of the importance of PARG during replication stress comes from ovarian cancer cells where loss of expression of key replication proteins (e.g., Timeless, Hus1 and RFC2), led to sensitivity to PARGi (Pillay et al., 2019).

Ray Chaudhuri et al. (2015) reported an increase in post replicative single stranded gaps following PARG depletion. This might be attributable to PARP1’s recently characterized function in sensing unligated Okazaki fragments (Figure 3) (Hanzlikova et al., 2018), and an as yet uncharacterised requirement for dePARylation for ligation to proceed. PARG depletion may therefore result in these PARylated Okazaki fragments being unresolved leading to post replicative single stranded gaps. Retention of XRCC1 at SSB sites is dependent on its BRCTII domain (Wei et al., 2013). Ligase III also contains a BRCTII domain and is involved in Okazaki fragment ligation. We hypothesize that ligase III could get trapped at PARylated Okazaki fragments, analogous to XRCC1 at SSBs (Figure 5).

Finally, PARP has also been shown to recruit XRCC1 to a subset of stalled (unresected) replication forks as a prerequisite for fork restart (Figure 3) (Ying et al., 2016). As above it is possible that PARG has a role in releasing PARP and XRCC1 so that fork restart can proceed (Figure 5), providing a second mechanism by which PARG depletion could lead to increased levels of reversed forks (Ray Chaudhuri et al., 2015). However, only a small reduction in stalled fork restart is seen in the absence of XRCC1 suggesting this function only mediates repair of a subset of stalled forks, perhaps where small lesions impede re-start of the forks.

In summary, evidence points to PARG having function in SSBR, DSBR and during replication (Figures 2, 3). It is likely that in many cases its role is to turn off PARP mediated function to ensure correct timing of the subsequent steps in each repair pathway. Thus, loss or inhibition of PARG will lead to altered DNA repair (Figure 5). Furthermore, if PARylation persists, the lesion formed is likely to act as a source of further DNA damage.

### Other Functions of PARG Contributing to PARGi-Mediated DNA Damage Sensitivity

There are additional mechanisms that could result in the observed DNA damage sensitization phenotypes. Activation of PARP following DNA damage leads to changes in chromatin structure due to PARylation of histones (Poirier et al., 1982). The negative charge of PAR decompacts the histone complex around chromatin, increasing its openness and accessibility; lack/inhibition of PARG could prolong this state and leave DNA more susceptible to DNA damage. In support of this, PARG null trophoblastic stem cells have been reported to keep chromatin decondensed and consequently increased the degree of intercalation by acridine orange and alkylation by MNNG and thymine base modifications into cyclobutane pyridine dimers induced by ultra violet (UV) light (Zhou et al., 2010; Koh, 2011). Thus, a function of PARG may be to limit the length of time chromatin is open, in order to maintain genomic stability.

Another function for PARG during DNA repair could involve regulating NAD+ consumption. Once activated by DNA damage, PARP uses cellular NAD+ as a substrate which is normally and rapidly recycled by PARG (Figure 1). However, upon excessive DNA damage, hyperactivation of PARP can cause cellular NAD+ and thus ATP to be depleted from cells and eventually result in mitochondrial membrane destabilization and the release of apoptosis inducing factors (AIF) to the nucleus (Yu et al., 2006; Stein and Imai, 2012). AIF translocation culminates in AIF-mediated apoptosis and DNA fragmentation (Yu, 2002) [reviewed in Robinson et al. (2019)]. Unsurprisingly given PARG’s role in PAR catabolism, loss of PARG exacerbates this form of AIF mediated caspase independent apoptosis named “parthanatos” (Zhou et al., 2011). Increased parthanatos has been reported in MNNG or UV treated PARG deficient backgrounds *in vitro* in breast cancer (Feng et al., 2012). This suggests following DNA damage another function of PARG is to suppress cell death.

### PARG in Mitosis

PARG depleted cells have evidence of mitotic defects, including fragmentation and amplification of centrosomes, multipolar spindles, chromosomal misalignment and aberrant segregation of chromosomes (Ame et al., 2009; Min et al., 2010). Further, PARG is enriched at spindles in Xenopus extracts (Chang et al., 2004). PARP1 null cells exhibit centrosome dysfunction and amplification (Kanai et al., 2003), and PARP1 and 2 localize to centromeres interacting with CENPA, CENPB, and Bub3 (Saxena et al., 2002). TNK1 and/or PARP3 depletion leads to spindle defects due to alteration in NuMA activity (Boehler et al., 2011). Thus PARG may co-operate with multiple PARP enzymes to regulate accurate mitosis. Consistent with this, after DNA damage PARGi led to spindle defects and the accumulation of cells at metaphase. Interestingly, TNK inhibition but not PARP1-3 inhibition, phenocopied the spindle defects (Gravells et al., 2018), highlighting the potential differences in mechanism of action of PARPi and PARGi.

## The Therapeutic Targeting of PARG

Like PARP, PARG depletion and inhibition are reported to have chemo and radiosensitisation effects (Table 3), in addition synthetic lethality has been reported in some contexts including HR deficiency. These observations are likely underpinned by effects on DNA repair and replication.

**TABLE 3:** Classes of DNA damage PARG depletion or inhibition alters cellular toxicity to.

**Class of DNA damage**	**Method of targeting PARG**	**Experimental system**	**Agent**	**References**
Alkylating agents	Homozygous deletion of PARG exons 2 and 3 (selective deletion of PARG110)	*In vivo*	MNU (N-Methyl-N-nitrosourea) Streptozotocin	Cortes et al., 2004
	PARG −/− mouse ES cells	*In vitro*	MNNG (methyl-nitronitrosoguanidine)	Koh et al., 2004
	PARGi (GPI 16552) in metastatic melanoma cancer cells	*In vitro* and *In vivo*	Temozolomide	Tentori et al., 2005
	PARGi (Gallotannin) in CHO cells	*In vitro*	MNNG	Keil et al., 2004
	PARG −/− mouse ES cells	*In vitro*	Dimethyl Sulfate	Fujihara et al., 2009
	PARG null mouse TS cells	*In vitro*	MNNG	Zhou et al., 2010
			Cyclo-phosphamide	
	Hypomorphic mutation of PARG leading to PARG110 −/− mouse embryonic fibroblasts	*In vitro*	MMS (methyl methanesulfonate)	Min et al., 2010
	PARG −/− ES Cells and siRNA in human MIAPaCa2 (pancreas) and RKO (Colon) cancer cell lines	*In vitro*	MMS	Shirai et al., 2013b
	PARGi (PDD00017273) in MCF7 breast cancer cells	*In vitro*	MMS	James et al., 2016
	PARGi – COH34 in various cancer cell lines	*In vitro*	Temozolomide	Chen and Yu, 2019
Cross linking agents	PARG −/− mouse ES cells	*In vitro*	Cisplatin	Fujihara et al., 2009
	PARG null mouse TS cells	*In vitro*	Cisplatin	Zhou et al., 2010
	PARG −/− ES Cells and siRNA in human MIAPaCa2 (pancreas) and RKO (Colon) cancer cell lines	*In vitro*	Cisplatin	Shirai et al., 2013b
	PARGi (COH34) in various cancer cell lines	*In vitro*	Cisplatin	Chen and Yu, 2019
	PARGi (PDD00017273) and shRNA in PDAC cell lines	*In vitro* and *in vivo*	Oxaliplatin	Jain et al., 2019
DNA metabolism	PARG −/− mouse ES cells	*In vitro*	Gemcitabine	Fujihara et al., 2009
	PARGi (PDD00017273) in ovarian cancer cell lines	*In vitro*	Gemcitabine	Pillay et al., 2019
			HU	
	PARGi – (PDD00017273) and shRNA in PDAC cell lines.	*In vitro*	5-Fluorouracil	Jain et al., 2019
Intercalators	PARG null mouse TS cells	*In vitro*	Epirubicin	Zhou et al., 2010
	PARGi (COH34) in various cancer cell lines	*In vitro*	Doxorubicin	Chen and Yu, 2019
Incorporated nucleotide analogs	PARG −/− mouse ES cells	*In vitro*	Gemcitabine	Fujihara et al., 2009
	PARGi (PDD00017273) in ovarian cancer cell lines	*In vitro*	Gemcitabine	Pillay et al., 2019
Oxidative damage	siRNA in MEFs	*In vitro*	Hydrogen peroxide	Blenn et al., 2006
	siRNA in A549 cancer cell line	*In vitro*	Hydrogen peroxide	Fisher et al., 2007
	Hypomorphic mutation of PARG leading to PARG110 −/− mouse embryonic fibroblasts	*In vitro*	Hydrogen peroxide	Min et al., 2010
Radiation	Homozygous deletion of PARG exons 2 and 3 (deletion of PARG110)	*In vivo*	γ-Irradiation	Cortes et al., 2004
	PARG−/− mouse ES cells	*In vitro*	γ-Irradiation	Fujihara et al., 2009
	siRNA in HeLa cells	*In vitro*	X-irradiation	Ame et al., 2009
	Hypomorphic mutation of PARG leading to PARG110 −/− mouse embryonic fibroblasts	*In vitro*	γ-Irradiation	Min et al., 2010
	PARG−/− mouse ES Cells	*In vitro*	γ-Irradiation	Shirai et al., 2013a
			Carbon ion irradiation	
			Fe-Ion Irradiaiton	
	siRNA, PARGi (PDD00017273) in MCF-7 breast cancer cell line	*In vitro*	γ-Irradiation	Gravells et al., 2018
	PARGi (JA2131) in prostate cancer PC3 cell line	*In vitro*	Irradiation	Houl et al., 2019
Topoisomerases inhibitors	PARG −/− mouse ES cells	*In vitro*	Camptothecin	Fujihara et al., 2009
	shRNA HeLa cell line	*In vitro*	Camptothecin	Ray Chaudhuri et al., 2015
	PARGi – COH34 in various cancer cell lines	*In vitro*	Camptothecin	Chen and Yu, 2019

*Black indicates sensitization by depletion or inhibition, red indicates no difference in response.*

### Radiosensitisation

*In vitro* reports in mouse embryonic stem cells and different cancer backgrounds where PARG has been silenced/depleted have consistently produced increased sensitivity to ionizing radiation (IR) (Ame et al., 2009; Min et al., 2010; Nakadate et al., 2013; Shirai et al., 2013a). This is thought to be underpinned by an increase in mitotic defects that culminate in mitotic catastrophe and cell death (Ame et al., 2009; Nakadate et al., 2013). These observations have been replicated with PARG inhibitors (Gravells et al., 2018; Houl et al., 2019). Tankyrase inhibition partially phenocopied PARGi promoting aberrant spindles and radiosensitisation suggesting PARGi may at least partially mediate its effects by preventing the reversal of tankyrase activity (Gravells et al., 2018). Both PARP and PARG inhibition delayed the resolution of IR induced RAD51 foci consistent with PARG reversal of PARP activity (Gravells et al., 2018). However, a report that TNK can PARylate CHK2 (Nagy et al., 2016) to promote HR raises the possibility that lack of reversal of TNK activity can also effect HR. Despite slower resolution of RAD51 foci, PARG inhibition increased the speed at which γH2AX foci were resolved compared to PARPi treated cells (Gravells et al., 2018). This suggests that the DNA damage induced by radiation is resolved quicker when PARG is inhibited. A possible explanation for the increased resolution was the increased IR-induced phospho-DNA-PK(S2056) foci reported under PARGi versus PARPi (Gravells et al., 2018). Increased numbers of DNA-PK foci can be indicative of an increase in cNHEJ, and PARG inhibition could therefore be functioning to promote cNHEJ by preventing reversal of PARP1/3 dependent PARylations (Figure 5). Alternatively, TNK is reported to PARylate DNA-PK to stabilize it (Dregalla et al., 2010) and it is possible that inhibition of PARG increases total cellular levels of DNA-PK altering the DNA repair equilibrium. Finally, phospho-DNA-PK(S2056) can also accumulate at unresected stalled replication forks (Ying et al., 2016), thus it is possible that PARG inhibition potentiates radiation induced stalled forks altering repair kinetics.

### Chemosensitisation

The reported chemosensitising effects of PARG depletion/deletion/inhibition are variable. The majority of the reports indicate sensitisation to different classes of DNA damaging agents (Table 3 and references there in). It is likely that the chemosensitizing mechanism is through reversal of PARP1-3 function in SSBR and DSBR or via effects on replication, but this has yet to be fully explored.

### Synthetic Lethality

PARG has been reported to be synthetically lethal (SL) with different genes that often undergo loss of function mutations in cancer, enabling targeted cell death. PARGi or PARG depletion has been observed to be synthetically lethal in BRCA2 depleted and deficient breast cancer cells (Fathers et al., 2012; Gravells et al., 2017). PARG inhibition caused an increase in replication fork collapse and replication associated DNA damage (Fathers et al., 2012; Gravells et al., 2017). Accordingly, increased levels of RAD51 foci and HR were also observed. Like PARPi, PARGi therefore seem to increase the reliance on HR for fork stability/restart, hence lethality in a BRCA2 mutant background (Fathers et al., 2012; Gravells et al., 2017). Synthetic lethality has also be reported with other HR related proteins including BRCA1, PALB2, FAM175A (ABRAXAS), and BARD1 in breast cancer cells where a more specific PARGi, PDD00017273, was used and on-target effects validated using two independent PARG siRNA (Gravells et al., 2017). Synthetic lethality between PARGi and BRCA1/2 was confirmed using PDD00017273 in pancreatic cancer cells (Jain et al., 2019) and with a second PARGi, COH34, in ovarian cancer cells (Chen and Yu, 2019). In contrast, siRNA mediated depletion of PARG in BRCA1 and PTEN deficient/proficient cells with a different genetic background has been reported to not be synthetically lethal (Noll et al., 2016). It is possible the nature of the BRCA1 deficiency may also be significant.

Interestingly, when screening ovarian cancer cell lines for sensitivity to the PARGi PDD00017273, cells that were differentially sensitive to PARG but not PARP inhibition were identified (Pillay et al., 2019). These cells had a replication catastrophe event upon PARGi (identified as pan-nuclear γH2AX staining) which was not seen with the PARPi olaparib. This suggests that low expression of key replication factors that promote fork stabilization, may be a biomarker predictive of PARGi effectiveness and that PARGi could be used as an alternative to PARPi. Consistent with this, BRCA1 mutated cells that had gained resistance to PARPi through loss of 53BP-1, were still more sensitive to the PARGi COH34 than BRCA1 wildtype cells (Chen and Yu, 2019). PARGi has also been reported to be synthetic lethal in XRCC1 depleted and deficient cells (Martin et al., 2018). This suggests PARGi may have efficacy in XRCC1 tumors; we can speculate that this is due to the function of XRCC1 in stabilizing stalled forks. Furthermore, PARG depletion via siRNA was reported to be synthetic lethal with dual specificity phosphatase 22 (DUSP22) via suppression of the mTOR/PI3k/AKT and an increase in the expression of PUMA inducing increased apoptosis in lung cancer (Sasaki et al., 2019). This requires validation with an inhibitor but suggests that PARGi in tumors deficient in DUSP22 are viable targets for PARGi and that other genetic targets that induce apoptosis may be worth investigating.

## The Role of PARG in Cancer

The therapeutic potential of targeting PARylation using PARP or PARG inhibitors alone or in combination with other therapies clearly has promise as an anti-cancer therapy. However, genetic manipulation of PARP and PARG suggests that PAR levels can also impact tumor induction and progression. The mechanisms by which changes in PARP expression can induce tumor formation and progression are well documented (reviewed in Schiewer and Knudsen, 2014). However, it is far less clear the role PARG plays having been reported to both promote (Dai et al., 2019; Marques et al., 2019) and suppress (Molloy-Simard et al., 2012) tumourigenesis. Genetic manipulation of PARG has demonstrated roles in proliferation (Pan et al., 2012), differentiation (Wang et al., 2019), metastasis (Li et al., 2012; Pan et al., 2012; Marques et al., 2019) and angiogenesis (Pan et al., 2012). It will be interesting to see if PARG inhibitors can recapitulate any of these findings as this will greatly increase their potential as cancer therapeutics.

### Roles Outside of DNA Repair

Genetic manipulation of PARG suggests that PAR levels can impact tumor induction and progression (Li et al., 2012; Molloy-Simard et al., 2012; Pan et al., 2012; Dai et al., 2019; Marques et al., 2019; Wang et al., 2019). Although changes in DNA repair capacity certainly contribute to genomic instability and therefore tumourigenesis, there are other likely impacts within the cell when PARG is disrupted. PAR also mediates a wide range of effects on transcription (reviewed in (Schiewer and Knudsen, 2014)). Studies have primarily focused on PARP1, however, altered PARylation as a result of changes in PARG expression is also likely to have a transcriptional effect. In support of this, PARG overexpression increased dePARylation of SMAD2/3, increasing SMAD target gene transcription which was in part responsible for the tumourigenic phenotype observed as a result of PARG expression (Dahl et al., 2014; Marques et al., 2019). In addition, PARG silencing decreased PARP1 and NF-kB expression which influenced DC and T cell fate to promote a more favorable CD4/CD8 ratio which could suppress tumor formation (Wang et al., 2019). When PARP and PARG depleted cells were transcriptionally profiled, each regulated both a unique set of genes, however, in each case there was also a similar number of overlapping genes. Interestingly for the common genes PARP and PARG acted in a similar, rather than opposing, fashion to regulate gene expression (Frizzell et al., 2009), suggesting that in this context they may work in concert.

The function of PARG in maintaining cellular NAD+ in the context of DNA repair has been discussed. In addition, altered NAD+ homoeostasis will likely compromise many aspects of metabolic signaling that influence tumor formation, transcription, behavior, and survival.

## Discussion

In conclusion, like PARP, PARG has multiple and complex roles in DNA repair and replication. Many are related to the reversal of autoPARylation of PARP1-3, but removal of PAR from other targets such as RECQ1 or TNK1/2 are emerging as important. In addition, it is likely that PARG impinges on many other aspects of tumor biology, via regulation of oncogenic signaling pathways. A number of pre-clinical studies now demonstrate that PARG inhibitors show promise as anti-cancer therapeutics, however, our understanding of the consequences of PARG inhibition need to further be refined and the identification of novel contexts in which PARG has promise as a target for inhibition need to be determined. Thus, despite more than 50 years of study, PAR biology continues to yield interesting and therapeutically significant results.

## Author Contributions

DH and HB conceived and drafted the publication. PG and RT contributed to the text and figures. All authors finalized the review, contributed to the article, and approved the submitted version.

## Conflict of Interest

The authors declare that the research was conducted in the absence of any commercial or financial relationships that could be construed as a potential conflict of interest.

## Abbreviations

altEJ: alternative non-homologous end-joining
ARH: ADP-ribose hydrolases
ART: (ADP-ribosyl)transferase
CHK2: checkpoint protein 2
DSBR: double strand break repair
DSBs: double strand breaks
HR: homologous recombination
HU: hydroxyurea
IR: ionizing radiation
MAR: mono(ADP-ribose)
NAD+: nicotinamide adenine dinucleotide
NHEJ: non-homologous end-joining
PAR: poly(ADP-ribose)
PARG: Poly(ADP-ribose) glycohydrolase
PARGi: PARG inhibitors
PARP: poly(ADP-ribose) polymerase
PARPi: PARP1/2/3 inhibitors
SAM: sterile alpha module
SSBR: single strand break repair
SSBs: single strand breaks
TIPs: tankyrase interacting proteins
TNK: Tankyrase.
